# Effect of ADHD medication on risk of injuries: a preference-based instrumental variable analysis

**DOI:** 10.1007/s00787-023-02294-6

**Published:** 2023-09-24

**Authors:** Tarjei Widding-Havneraas, Felix Elwert, Simen Markussen, Henrik Daae Zachrisson, Ingvild Lyhmann, Ashmita Chaulagain, Ingvar Bjelland, Anne Halmøy, Knut Rypdal, Arnstein Mykletun

**Affiliations:** 1 Centre for Research and Education in Forensic Psychiatry, Haukeland University Hospital, Bergen, Norway; 2 Department of Clinical Medicine, University of Bergen, Bergen, Norway; 3 Department of Sociology, University of Wisconsin-Madison, Madison, WI USA; 4 Department of Biostatistics and Medical Informatics, University of Wisconsin-Madison, Madison, WI USA; 5 Ragnar Frisch Centre for Economic Research, Oslo, Norway; 6 Department of Special Needs Education, University of Oslo, Oslo, Norway; 7 Division of Psychiatry, Haukeland University Hospital, Bergen, Norway; 8 Division of Health Services, Norwegian Institute of Public Health, Oslo, Norway; 9 Department of Community Medicine, University of Tromsø, Tromsø, Norway; 10 Centre for Work and Mental Health, Nordland Hospital, Bodø, Norway

**Keywords:** ADHD, Pharmacological treatment, Injury, Quasi-experiment, Instrumental variable

## Abstract

ADHD is associated with an increased risk of injury. Causal evidence for effects of pharmacological treatment on injuries is scarce. We estimated effects of ADHD medication on injuries using variation in provider preference as an instrumental variable (IV). Using Norwegian registry data, we followed 8051 patients who were diagnosed with ADHD aged 5 to 18 between 2009 and 2011 and recorded their ADHD medication and injuries treated in emergency rooms and emergency wards up to 4 years after diagnosis. Persons with ADHD had an increased risk of injuries compared to the general population (RR 1.35; 95% CI: 1.30–1.39), with higher risk in females (RR 1.47; 95% CI: 1.38–1.56) than males (RR 1.23; 95% CI: 1.18–1.28). The between-clinics variation in provider preference for ADHD medication was large and had a considerable impact on patients’ treatment status. There was no causal evidence for protective effects of pharmacological treatment on injuries overall for young individuals with ADHD characterized by milder or atypical symptoms. However, there was an apparent effect of pharmacological treatment over time on the risk of injuries treated at emergency wards in this patient group.

**Supplementary Information:**

The online version contains supplementary material available at 10.1007/s00787-023-02294-6.

## Introduction

Injuries are the worldwide leading cause of death and disability among children and adolescents [1, 2]. Meta-analyses have found that youth with attention-deficit/hyperactivity disorder (ADHD) have a higher risk of injuries compared to those without ADHD [3, 4]. Additionally, people with ADHD have a heightened risk of suicide attempts [5], suicide, and injury-related death [6, 7]. The increased injury risk in ADHD have been attributed to the core ADHD symptoms of impulsivity, hyperactivity, inattention, and common comorbid disorders such as conduct disorder (CD) and oppositional defiant disorder (ODD) [3]. Consequently, injury prevention is especially important for this high-risk group.

Randomized controlled trials (RCT) show that ADHD medication reduces short-term ADHD symptoms [8], but no similar results exist from RCTs for reduction in injuries. Meta-analytic evidence suggests that ADHD medication can reduce injuries [3, 9, 10]. ADHD medication is associated with reductions in emergency room visits [11], traumatic brain injuries [12], burn injuries [13], bone fractures [14], transport accidents [15], all-cause mortality [16], with mixed evidence for suicide attempts [17]. There is less knowledge about treatment effects in children and adolescents [18]. Moreover, geographical variation in diagnosis and treatment of ADHD have led to concerns about under- and overtreatment caused by clinical practice variation [19–22]. There are calls for more knowledge about treatment effects among persons who may receive treatment due to varying clinical practice which likely concerns patients with milder symptoms [23]. Such knowledge can be obtained by using a quasi-experimental provider preference IV design combined with population-wide data with several years follow-up.

We use idiosyncratic variation in provider preference for pharmacological treatment across clinics as an instrumental variable (IV) to identify causal effects of pharmacological treatment of ADHD on the risk of injuries among patients on the margin of treatment. Between-clinics variation in provider preference represent a source of “as good as” randomization to treatment for these patients and we thus circumvent unmeasured confounding and obtain treatment effects for a clinically relevant population [24, 25]. Only two other studies have used provider preference as an IV for effects of ADHD medication on injuries. A Danish study finds protective effects of medication on hospital visits that may be driven by a reduction in injuries, although estimates are imprecise [26]. Similarly, a US Medicaid claims-based study finds that ADHD medication reduces the yearly incidence of injuries and injury spending [27]. Thus, more causal knowledge is needed about treatment effects on long-term functional outcomes, such as injuries, and in particular among persons who may be treated differently due to varying clinical practice [23, 28–30].

The main aim of this study is to estimate the effect of pharmacological treatment of ADHD on injuries for patients on the margin of treatment by use of such a design. We use registry data for the entire Norwegian population to estimate the causal effect of ADHD medication injuries up to four years following diagnosis through a provider preference IV design.

## Methods

### Sample

Our ADHD patient sample includes all patients who were diagnosed with ADHD for the first time between the ages of 5 and 18 in 2009–2011 (*n* = 8,051) by the Norwegian Child and Adolescent Mental Health Services (CAMHS), as registered in the Norwegian Patient Registry (NPR). The ADHD patient sample consists of persons diagnosed with ICD-10 Hyperkinetic disorder, i.e., F90.0 (81.3%), F90.1 (11.3%), F90.8 (6.2%), and F90.9 (1.1%). Additionally, we constituted a general population comparison sample aged 5–18 without contact with CAMHS in 2009–2011 that were assigned a randomly generated inclusion date in 2009–2011 (*n* = 75,184).

### Injuries

Injuries include intentional and unintentional accidental or self-inflicted physical damage caused by sudden or cumulative transfers of energy [31]. We used data on all contacts for injuries treated at emergency rooms (ER) in primary care (mainly outpatient clinics) registered in the Norwegian Control and Payment of Health Reimbursements Database (KUHR) and emergency wards (EW) in secondary care (i.e., hospitals) registered in NPR. Contacts at ER are coded according to the International Classification of Primary Care, 2nd edition (ICPC-2). We defined cumulative indicators for any injury-related contact at ER or EW taking value one if registered with an injury code, and zero otherwise, separately for each of the first four years following diagnosis. We defined three primary outcomes: any injuries at either ER or EW, only ER, and only EW. For ER-related contacts, we also defined a set of indicators for types of injuries by body part based on a categorization developed by the Norwegian Institute of Public Health: head, fracture, sprain, burn, poison, penetration, ear, eye, other (ICPC-2 codes in Table S1), also including suicide-related contacts. EW-related contacts included contacts for injuries, self-harm, or violence/assault.

### ADHD medication

We used data for filled ADHD prescriptions from the Norwegian Prescription Database for ADHD medications as defined by the Norwegian Institute of Public Health (percent of total ADHD prescriptions in parenthesis). Stimulants included Metylphenidate (N06BA04, 87.5%), Dexamphetamine (N06BA02, 0.8%), Lisdexamfetamine (N06BA12, 0.06%), Amphetamine (N06BA01, 0.04%), while non-stimulants included Atomoxetine (N06BA09, 11.54%). Pharmacological treatment was defined as the cumulative number of defined daily doses (DDD) filled for any ADHD prescription over one to four years after being diagnosed with ADHD. Treatment was scaled to make one unit increase correspond to an increase from 0 to full-time pharmacological treatment over follow-up. Intuitively, then, the treatment effect can be interpreted as the contrast in the risk of injury between no ADHD medication during follow-up vs. ADHD medication corresponding to full-time follow-up (e.g., 0 vs. 365 DDD by one year follow-up).

### Covariates

We included covariates for patients, their families, and the clinics’ catchment area to adjust analyses for patient mix and catchment area characteristics. Patient covariates were measured at baseline and catchment area covariates was measured between 2009 and 2011. The following variables were adjusted for: age, sex, comorbid diagnosis at time of diagnosis, country of birth (Norway, Europe, Outside Europe), year of contact with clinic, injuries prior to ADHD diagnosis, child protection service intervention prior to ADHD diagnosis, and parents’ labor income and highest education when the child was six-years (primary school, high school, short- and long university education) and marital status (married, unmarried, other (widowed, divorced, separated)). Catchment area characteristics included population size, high school dropout rates and, using aggregated measures from the general population sample: percent of youth immigrants, parents’ labor income, parents’ education level, mother’s marriage rate (overview of data sources, Table S2).

### Statistical analyses

We computed risk ratios for any injury and types of injuries at 4 years follow-up for patients with ADHD relative to the matched sample with generalized linear models. Linear probability models (LPM) were used to estimate associations between pharmacological treatment and injuries [32]. The estimand is the average treatment effect on the treated (ATT). Causal interpretation of LPM estimates requires that the exposure is assumed to be conditionally random given covariates [33]. This is unlikely and motivates our IV design. Analyses were conducted on multiple samples: all patients and stratified by sex due to potentially important differences in ADHD and injury, by stimulants/non-stimulants as effectiveness may differ, and in patients aged 5–12 and 13–18 at time of diagnosis (median-split).

The IV design used the observed variation in pharmacological treatment between clinics as quasi-randomization to pharmacological treatment net of patient-mix [24]. Consider two similar patients at two clinics with varying treatment preference: one patient is not treated while the other is treated due to a stronger treatment preference. Treatment effects, then, concern patients on the margin of treatment, leaving out patients where there is strong clinical consensus on treatment [27]. Individuals with ADHD in a patient role at the margin of medication is not a clearly defined group. However, the phenomenon and cases are recognizable by ambivalence in medication decisions, or by the awareness that colleagues would reach other decisions on medication. The estimand is the local average treatment effect (LATE), which is the average treatment effect among patients on the margin for pharmacological treatment who receive treatment due to their provider’s preference [32].

In the Norwegian healthcare system, pharmacological treatment initiation is within the discretion of psychiatrists who collaborate in teams at clinics. To measure provider preference, we calculate the average number of DDD for filled ADHD prescriptions for patients with ADHD at clinic level. We selected a four-year time frame as the IV was sufficiently strong only during these years. We show medication over an eight-year period in Fig. 2B to illustrate the long-term development. Provider preference was measured as a leave-one-out average to exclude any potential impact an individual patient may have on the preference they are exposed to. The IV was scaled in the same manner as the treatment. IV analyses were conducted on the same samples as LPM. IV rely on the important assumptions [24, 34]. Relevance is tested with the *F*-statistic from the first stage. Exclusion is examined by reduced form analyses based on the general population sample. Independence is examined with tests of covariate balance over values of the IV. Monotonicity is investigated by examining the association between treatment and provider preference (more details, Supplementary Sect. 1.2). Estimation of LATE was based on two-stage least squares (2SLS). As robustness checks, we estimated models using Probit [35, 36]. We also examined robustness of results by excluding a subset of patients who had filled prescriptions prior to their sample inclusion date. Standard errors were clustered by clinics. All analyses were done in Stata 17 [37] and coefficient plots was made with *coefplot* [38]. We followed reporting guidelines for IV analyses [39] and preregistered (ISRCTN: 11891971) and protocolled our analyses [30].

## Results

### Descriptive statistics

Table 1 shows baseline characteristics of the ADHD patient sample and the general population sample. The ADHD sample had more males, Norwegian background, and injuries before inclusion. Parents of patients with ADHD had lower income, education, and marriage rate. Catchment area characteristics were relatively similar.

**Table 1:** Baseline characteristics for patients with ADHD and the general population, aged 5–18 in 2009–2011 (*n* = 83,235)

	ADHD diagnosis when in contact with CAMHS 2009–11 (*n* = 8051)	General population, excluding those in contact with CAMHS 2009–11 (*n* = 75,184)
Patient characteristics		
Age at diagnosis, mean ± SD^1^	11.7 ± 3.4	11.6 ± 4
Male, no. (%)	5566 (69.1)	38,505 (51.2)
Country of birth, no. (%)		
Norway	6263 (77.8)	52,618 (70.0)
Europe	1080 (13.4)	11,204 (14.9)
Outside Europe	707 (8.8)	11,362 (15.1)
Injury before diagnosis, no. (%)	4768 (58.2)	34,469 (45.9)
Child protection service before diagnosis, no. (%)	1379 (17.13)	1614 (2.2)
Comorbidity, no. (%)	2003 (24.9)	–
Family characteristics		
Parents’ labor income (USD), mean ± SD^2^		
Labor income, father	54,900 ± 40,410	69,311 ± 66,870
Labor income, mother	28,374 ± 24,879	35,929 ± 29,999
Parents’ highest education, no. (%)		
University long, father	316 (3.9)	8143 (10.8)
University short, father	994 (12.4)	15,859 (21.1)
High school, father	3849 (47.8)	33,673 (44.8)
Primary school, father	2561 (31.8)	14,028 (18.7)
University long, mother	221 (2.8)	5398 (7.2)
University short, mother	1629 (20.2)	23,549 (31.3)
High school, mother	3437 (42.7)	28,264 (37.6)
Primary school, mother	2640 (32.8)	15,031 (20.0)
Parents’ civil status, no. (%)		
Unmarried, father	2356 (29.3)	15,432 (20.5)
Married, father	3767 (46.8)	46,622 (62.0)
Other, father	1474 (18.3)	9050 (12.0)
Unmarried, mother	2526 (31.4)	16,503 (22.0)
Married, mother	3785 (47.0)	46,549 (61.9)
Other, mother	1604 (19.9)	9829 (13.1)
Catchment area characteristics		
Youth immigrants, % ± SD	26.8 ± 10.5	30.0 ± 13.0
Parents’ primary school education, % ± SD	7.9 ± 4.6	9.0 ± 6.0
Parents’ married, % ± SD	60.4 ± 6.3	61.6 ± 6.0
Parents’ labor income (USD), mean ± SD	48,019 ± 7,192	49,858 ± 9,726
High school dropout, % ± SD	25.6 ± 4.1	24.8 ± 4.3
Population (0–65 + yrs.), mean ± SD	32,913 ± 26,765	37,696 ± 30,506

^1^Plus-minus values are mean ± SD. ^2^USD/NOK exchange rate average for 2010 (USD 1/NOK 6.0453)

**Fig. 1:**
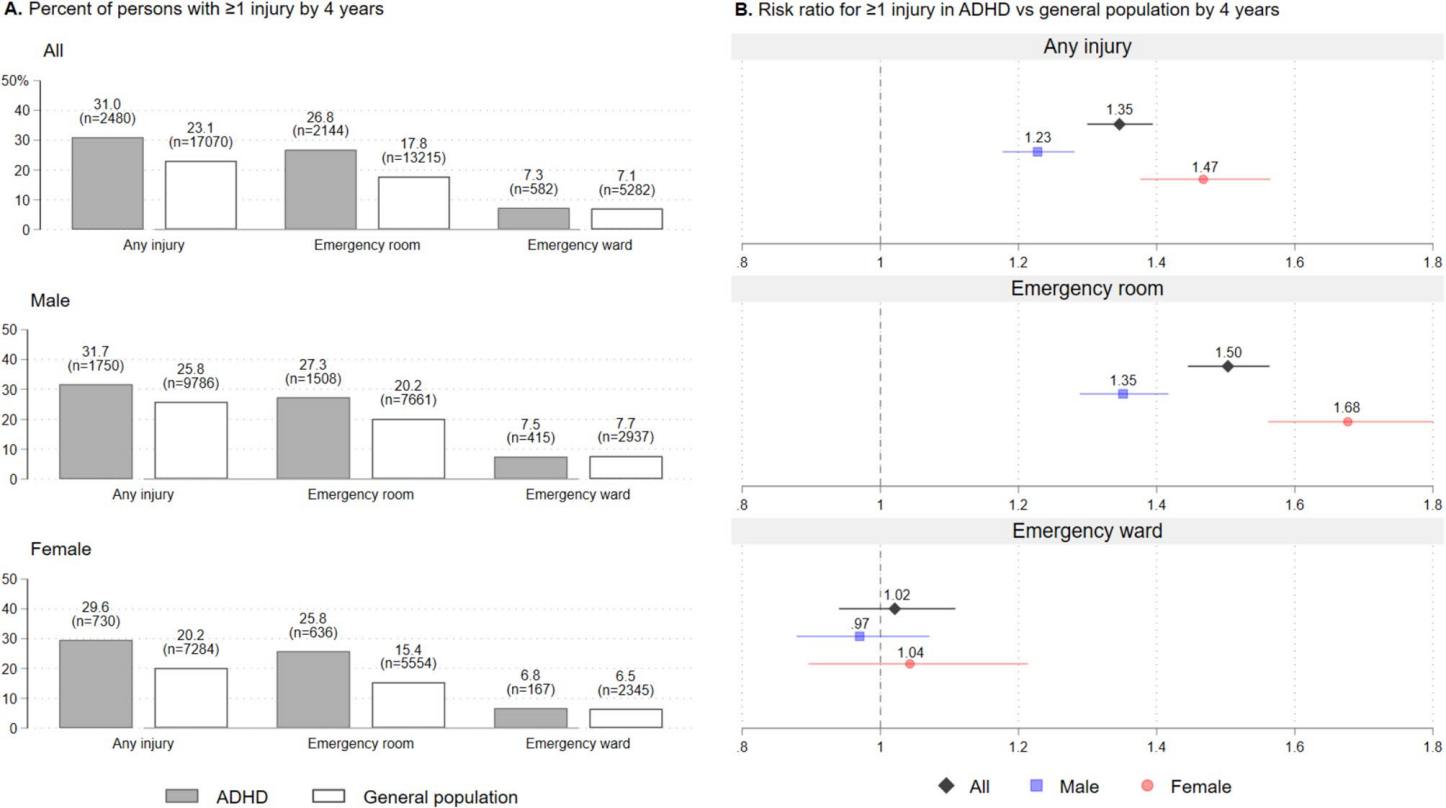
ADHD, general population, and risk of injuries by four years follow-up after 2009–2011. Patients diagnosed with ADHD in 2009–2011 and general population excluding those in contact with child and adolescent mental health services in 2009–2011 aged 5 to 18 at time of inclusion (unique *n* = 83,235) excluding those who either died (*n* = 48) or emigrated (*n* = 1091), and percentage reported for each bar

Figure 1 shows higher rates of any injury and injury contacts at ER, but not EW, for both male and female patients with ADHD compared to the general population over four-years follow-up. The highest risk ratios were for injuries treated at ER. Patients with ADHD and comorbid CD/ODD had somewhat higher prevalence of any injuries (37.2%) at 4 years follow-up. In terms of specific types of injuries, persons with ADHD had higher risk of all types of injuries with the ER, except for burn injuries (Figure S1). The highest increased risk was for suicide-related contacts with ER, followed by self-harm and victimization-related contacts with EW (Figure S2). Except violence-related injuries, the increased risk was highest for penetration-, poison-, and ear-related injuries. There was, however, relatively few events related to self-harm-, victimization-, poison-, and ear-injuries.

### Evaluation of instrumental variable

**Fig. 2:**
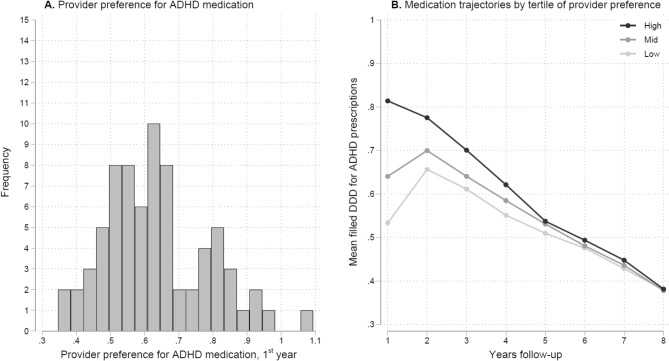
Variation between clinics in pharmacological treatment of ADHD among patients diagnosed with ADHD. **A** Provider preference for pharmacological treatment at clinic level as mean defined daily doses (DDD) for ADHD medication first year after patients’ ADHD diagnosis on *x*-axis. **B** Providers’ pharmacological treatment trajectories. Yearly mean filled DDD for ADHD prescriptions after diagnosis scaled so 1 equal 365 DDD and divided into tertiles (high, mid, low) of clinics first year prescription preference

Figure 2A shows the distribution of provider preference measured as DDD for filled ADHD prescriptions scaled by 365 (i.e., a value of 1 corresponds to 365 DDD). Median DDD was 0.65 (interquartile range: 0.25; coefficient of variation: 0.22). Clinics had a median of 77 patients (interquartile range: 90). 78.1% of all patients with ADHD had filled ≥ 1 ADHD prescription the first year after diagnosis, 87.5% by four years, and 89.9% by eight years follow-up. Figure 2B shows that variation in provider preference varies from 0.53 in the lowest to 0.81 in the highest tertile in the first year of follow-up, and subsequently converges to 0.55–0.62 by 4 years follow-up, and 0.38 in both tertiles by 8 years follow-up. Prescription rates remained consistently highest and lowest in the upper and lower tertile, and converged to similar values by five years. The P90/P10 ratio was 1.79 the first year and 1.65 by 4 years follow-up. Relevance is supported by strong first stage *F*-statistics above the conventional threshold of 10, with year one to three above the recent suggested threshold of 104.7 [40]. The *F*-statistic for year one to four was 460.3, 217.3, 139.4, and 88.7 (Figure S3). The balance of covariates across the IV was relatively strong as shown by low joint *F*-statistic values (Figure S4). Provider preference was not associated with injury in the general population, supporting exclusion (Figure S5), and had a monotonic relationship with medication (Figure S6).

**Fig. 3:**
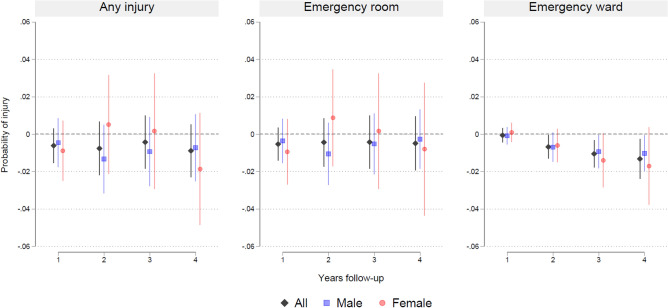
Associations between ADHD medication and injuries from linear probability models. Patients with ADHD diagnosis in Norway 2009–2011 aged 5–18 at time of diagnosis. Coefficient plots for regressions with 95% confidence intervals from LPM adjusted for patient mix

Figure 3 presents associations between pharmacological treatment and the probability of any injuries, injuries in ER and EW from LPMs for 1–4 years follow-up after ADHD diagnosis for all patients and by sex. There was no evidence of associations between pharmacological treatment and any injuries nor injuries treated at ERs. There was support for negative association between treatment and injuries at EWs at three-years follow-up overall (−1.0 percentage point (pp.), 95% CI -1.8 to −0.3) and for females (−1.4 pp., 95% CI −2.8 to 0.04) and four-years follow-up overall (−1.3 pp., 95% −2.4 to −0.3) and for females (−1.7 pp., 95% CI −3.8 to −0.04). Probit models provided similar results (Figure S6). There were also similar results in subgroups of persons aged below and above the median age of 12 (Figure S7). Analyses of associations by medication type showed support for the same negative association between medication and EW, while there was no support for any associations for nonstimulant medication (Figure S8). Injury-specific LPM results are reported in the supplementary (Figure S9).

### Results for linear probability models and instrumental variable analyses

**Fig. 4:**
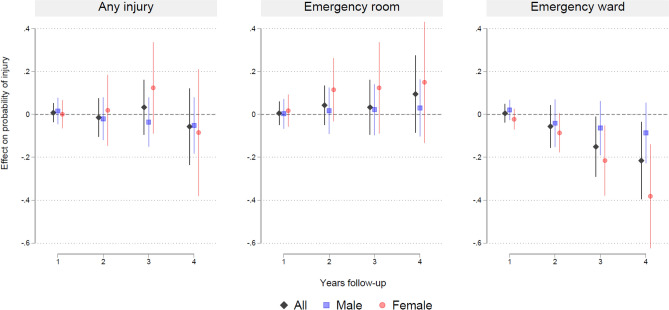
Effect estimates of ADHD medication on injuries from instrumental variable analyses. Patients with ADHD diagnosis in Norway 2009–2011 aged 5–18 at time of diagnosis. Coefficient plots for regressions with 95% confidence intervals. Two stage least squares estimates adjusted for patient mix

Figure 4 presents estimates of LATEs from 2SLS IV models for all patients and by sex. Treatment effects were relatively imprecise with wide 95% confidence intervals. The estimated treatment effects showed no evidence of pharmacological treatment on any injuries or injuries treated in ERs. There was support for pharmacological treatment reducing the probability of injuries in EW at three-years follow-up for all (−15.1 pp., 95% CI: −29.1 to −1.1) and at four-years follow-up for all (−21.6 pp., 95% CI: −39.5 to −3.7), which equals a number needed to treat (NNT) of 7 and 5, respectively. There was support for protective effects of medication on EW for females at 3 years follow-up (−21.5 pp., 95% CI: −37.8 to −5.3; NNT: 5) and 4 years follow-up (−38.2 pp., 95% CI: −62.3 to −14.0; NNT: 3). Robustness checks showed similar results, including models based on IV Probit estimation (Figure S10) and robustness analysis excluding patients who had filled one or more prescription prior to diagnosis (Figure S11).

There was no evidence of age-related variation in treatment effects (Figure S12). Results for IV analyses for stimulant medication were similar to the main IV analyses, while there was no support for any effects for nonstimulant medication (Figure S13). IV analyses for specific types of injuries indicated that pharmacological treatment reduced ER-related burn-injuries for all at two- (−2.6 pp., 95% CI: −4.1 to −1.1; NNT: 38) and 3 years (−3.0 pp., 95% CI: −5.3 to −0.7; NNT: 33) follow-up, and for males at two- (−3.0 pp., 95% CI: −5.1 to −0.9; NNT: 33), three- (−3.8 pp., 95% CI: −6.8 to −0.9; NNT: 26), and four-years (−4.4 pp., 95% CI: −7.8 to −0–9; NNT: 23) follow-up. There was no evidence for protective effects on other ER-related injury types (Figure S14).

## Discussion

### Main findings

This study estimated effects of pharmacological treatment of ADHD on injuries based on a preference-based IV design and population-wide registry data. While persons with ADHD had higher risk of injuries compared to the general population, we did not find clear evidence to support negative associations between pharmacological treatment and injuries in LP-regressions, although there was some support for EW-related injuries. Nonetheless, these results are likely affected by unmeasured confounding which we corrected for in IV analysis. There was large between-clinics variation in rates of pharmacological treatment which influenced patients’ treatment and there was support for the main underlying IV assumptions. IV analyses showed no causal evidence for protective effects of pharmacological treatment on injuries overall for patients on the margin of treatment. There was, however, an apparent effect of pharmacological treatment on the risk of emergency ward-related injuries in this patient group.

### Findings in context

Our findings support research showing that patients with ADHD are more prone to injuries than the general population. The overall RR of 1.35 (95% CI: 1.30–1.39) for any injury in persons with vs. without ADHD is similar to meta-analytic evidence [3]. The highest incidence of injuries were in males relative to females in line with existing knowledge [41]. However, females with ADHD had a higher risk of injuries than males with ADHD, which also supports existing research [42] and a potential reason may be that ADHD is more severe when detected among females in young age [43]. We contribute with analysis showing that people with ADHD have an increased risk of multiple types of injuries in both primary and secondary care, including suicide-related contacts, self-harm, and victimization. The findings that both self-harm and victimization is overrepresented in ADHD contributes to a topic with scarce high-quality data concerning a small but clinically important subgroup. There was no clear evidence of treatment effects in estimates of the average treatment effect on the treated (ATT) from LP-regressions. These estimates are likely biased upwards as patients with severe ADHD symptoms may be more likely to select positively into both treatment and injury.

We present novel causal evidence of effects of pharmacological treatment of ADHD on injuries in both primary and secondary care for patients on the margin of treatment. These effect estimates are relevant to clinical practice as they are informative for decision-making for patients where clinicians may come to varying conclusions about treatment, although such patients may be difficult to identify for the individual clinician in practice [44].

We found no support for protective effects of ADHD medication for overall nor ER-related injuries, which can be attributed to several factors. First, patients on the margin of treatment have milder symptom severity contributing to uncertainty about medication benefits, and they may also experience lower treatment effectiveness. Thus, it is important to consider that the treatment effects concern a subgroup of patients excluding individuals with the most severe ADHD symptoms. Second, the treatment effects were imprecise despite using a large nationwide sample with a strong IV and support for the main underlying assumptions. As the estimates were imprecise, we cannot rule out that smaller treatment effects remained undetected.

While there are apparent protective effects of ADHD medication on EW-related injuries for all and females after three and four-years (Fig. 3), these findings warrant cautious interpretation as the statistically significant negative estimates for injuries treated in EWs are estimated imprecisely and contrast with positive (but statistically insignificant) estimates for injuries treated in ERs.

Our findings of large but imprecisely estimated negative long-term effects on injuries treated in EW align with a Danish IV study [26] that found large but imprecisely estimated protective effects for young patients diagnosed with ADHD on the margin of treatment and a US-based IV study for the same patient group [27]. Similarly, our IV results that indicate protective effects on burn-related injuries are in line with a Taiwanese within-subjects study concerning all treated [13]. Generalization across national and institutional contexts as well as study populations warrant caution.

### Strengths and limitations

There are several strengths to this study. The combination of quasi-experimental IV design, extensive scrutiny of IV assumptions with statistical tests and subject matter knowledge, and comprehensive nationwide data produces treatment effects with a credible causal interpretation. The findings from the IV analysis have relevance for clinical practice as they provide evidence on long term pharmacological treatment effects for patients with clinical uncertainty.

Our study is situated within the context of the Norwegian universal healthcare system, which assigns patients to clinics based on their place of residence and has a negligible private sector. As in the US [45], considerable geographical variation in ADHD diagnoses and medication [20, 30] and clinicians’ attitudes toward ADHD [21] suggest practice variation. Provider preference is a more plausible IV after adjusting for patient mix, which we addressed with a rich set of covariates. To our knowledge, only one other study has combined a provider preference IV design with nationwide registry data to estimate the effects of pharmacological treatment for ADHD on health-related outcomes, namely any hospital contact and EW contacts [26].

There are limitations that should be considered. First, there are uncertainties tied to the IV design. Variation in provider preference needs to be random (conditional on covariates) for patients and the variation needs to affect only variation in pharmacological treatment. We adjusted for many variables but cannot rule all potential instrument-outcome confounding [46]. Geographical variation in ADHD symptom load is likely not a concern [20]. Second, clinics’ preference for psychosocial treatment may vary meaning that there could be more than one treatment and this could not be ruled out due to lack of appropriate data. However, receipt of pharmacological treatment may simultaneously indicate closer follow-up with clinics. Third, due to lack of sibling data and the high heritability of ADHD [47], we could not rule out siblings as a potential source of interference. Fourth, clinicians weigh risks and benefits in their treatment decisions and hence monotonicity may be violated in some settings [48]. However, our results supported a monotonic association between treatment and provider preference. Fifth, our sample is too small to detect precise treatment effects. Sixth, the use of filled prescriptions may include measurement error. Seventh, we cannot check whether persons in the sample filled prescriptions prior to 2009. Eight, data on injuries may be underreported as the data we used requires persons to seek help for their injuries [49]. Due to how Norwegian injury data are registered, there is no definitive way of ensuring that the same injury may be treated in both ER and EW, where the most common injuries include severe fractures, poisonings and head injuries [49]. As well, the largest EW units in the capital (Oslo) had higher registration quality the first years of the registry. However, any geographical bias would affect persons regardless of treatment status. Finally, diagnosis and medication of ADHD have increased considerably in Norway since our cohort was diagnosed with ADHD in 2009–2011 [8, 50, 51]. Since our study speaks to treatment effects in this group, our study may speak to a larger patient group today. Future research should investigate the implications of increasing diagnosis and medication rates for the long-term effect of pharmacological ADHD treatment on injuries.

## Conclusion

Our study highlights that persons with ADHD are a high-risk group for injuries and underscores the need to alleviate the burden of injury among these persons. We found no causal evidence of protective effects of pharmacological treatment of ADHD on the risk of injuries overall among patients on the margin of treatment. However, there was apparent protective effects of pharmacological treatment of ADHD on emergency ward related injuries, but these estimates were imprecise and warrant cautious interpretation. As such, the overall findings indicates that a possible protective effect on injuries is an invalid argument for pharmacological treatment of ADHD in patients on the margin of treatment. Nonetheless, there may be other valid arguments for such treatment among these patients. Additional studies on injury-related and other long-term outcomes should be conducted to improve our evidence base for treatment effects.

### Supplementary Information

Below is the link to the electronic supplementary material.

Supplementary file1 (PDF 1134 KB)

## Data Availability

The data cannot be made public due to data privacy laws. Data availability requires approvals from authorized data owners.
