# Supplementary material for: Effect of ADHD medication on risk of injuries: a preference-based instrumental variable analysis
Source: Eur Child Adolesc Psychiatry. 2023 Sep 24;33(6):1987–96. doi: 10.1007/s00787-023-02294-6 (PMC11211136; doi:10.1007/s00787-023-02294-6)
Supplement: Supplementary file 1 — Supplementary file1 (PDF 1134 KB) [file 787_2023_2294_MOESM1_ESM.pdf]

Online supplementary to

Effect of ADHD Medication on Risk of Injuries:

A Preference-Based Instrumental Variable Analysis

1.1 Data

Category	ICPC-2 codes
Head	N79, N80
Fracture	L72, L73, L74, L75, L76
Sprain	L77, L78, L79, L80, L81, L96
Burn	S14
Poison	A84, A86
Penetration	S13, S18
Ear	H76, H77, H78, H79
Eye	F75, F76, F79
Other	S12, S15, S16, S17, S19, A80, A81,
	A88, B76, B77, D79, D80, N81,
	R87, R88, U80, X82, Y80
Suicide	P77

Table S1. Categories of injury with ICPC-2 codes. The category “Other” combines “other” and “other surface injuries”.

Covariates	Data source
Patients	
Age	Norwegian Patient Registry
Sex	Norwegian Patient Registry
Year of contact	Norwegian Patient Registry
Comorbidity	Norwegian Patient Registry
Country of birth	Central Population Registry
Injury before diagnosis/inclusion	Central Reimbursement and Norwegian Patient Registry
Emigration	Central Population Registry
Death	Norwegian Cause of Death Registry
Family	
Parents labor income	Income, Tax, and Wealth Registry
Parents education level	Norwegian Education Database
Parents marital status	Central Population Registry
Catchment area	
Youth immigration	Central Population Registry
Parents labor income	Income, Tax, and Wealth Registry
Parents education level	Norwegian Education Database
Parents marital status	Central Population Registry
High school dropout rate	Statistics Norway
Population	Statistics Norway

Table S2. Data sources for covariates.

1.2 Instrumental variable analysis

Provider preference need to meet the following requirements to be considered a valid IV.[1-3] First, provider preference must predict treatment (relevance). This is tested with the F-statistic of the IV in first stage regressions. Second, provider preference can only impact injuries by its effect on treatment (exclusion). This was assessed by reduced form analyses in the general population. Third, provider preference must be as good as random for patients (independence), which we account for by including covariates for patient mix. Fourth, provider preference can only impact patients' treatment either positively or negatively (monotonicity), which is examined by analyses of the relationship between provider preference and medication. Fifth, there should be no interference nor treatment variation (stable unit treatment value assumption) which we assess through analyses of medication type.

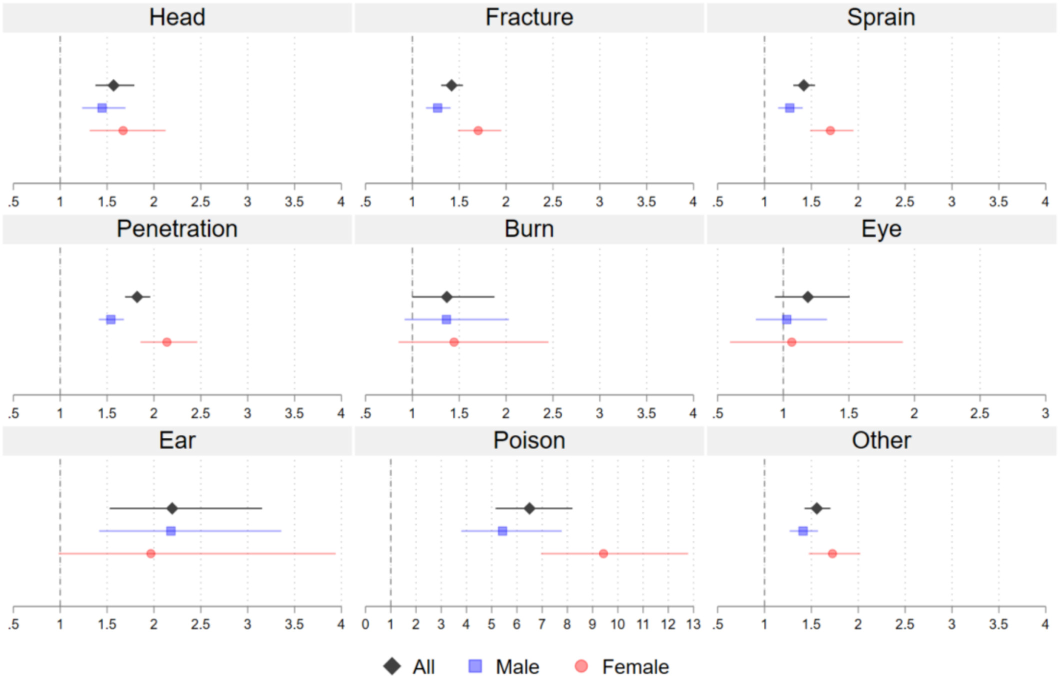

Figure S1. **Risk ratios for specific types of injuries at emergency room in persons with ADHD vs general population by 4 years follow-up.** *x*-axis differs for poison due to large estimates (but also low frequency of events).

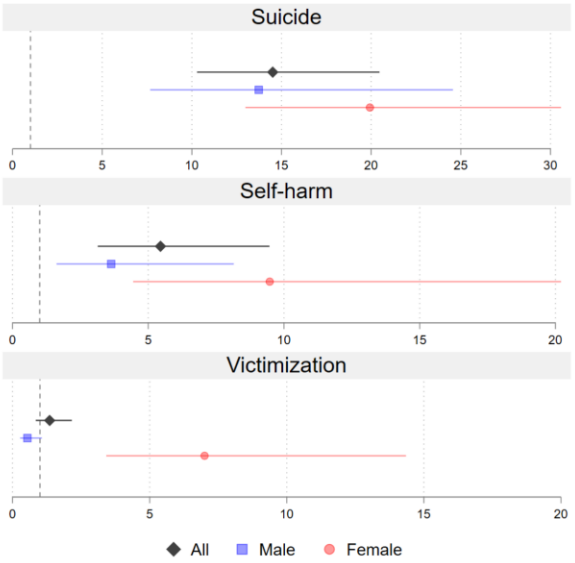

Figure S2. Suicide, self-harm, victimization. Suicide-related contacts at ER and self-harm- and victimication-related contacts at EW.

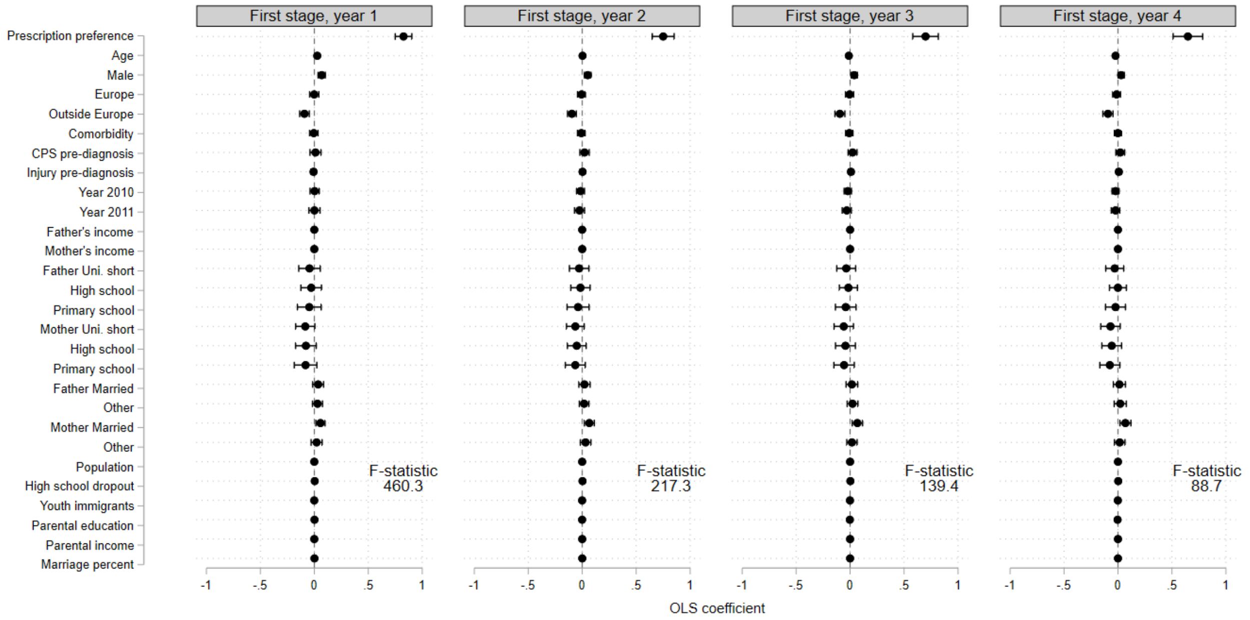

Figure S3. Coefficient plot for first stage results with *F*-statistics for the IV.

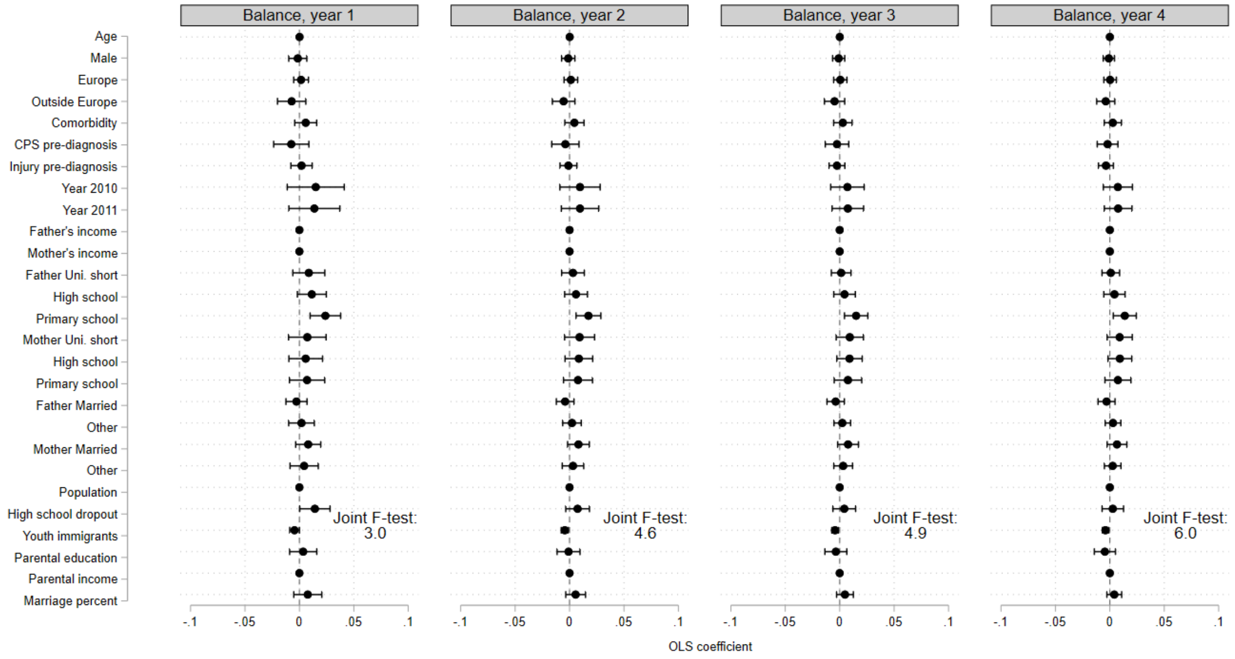

Figure S4. Coefficient plot examining balance of covariates for the IV with the joint F-test.

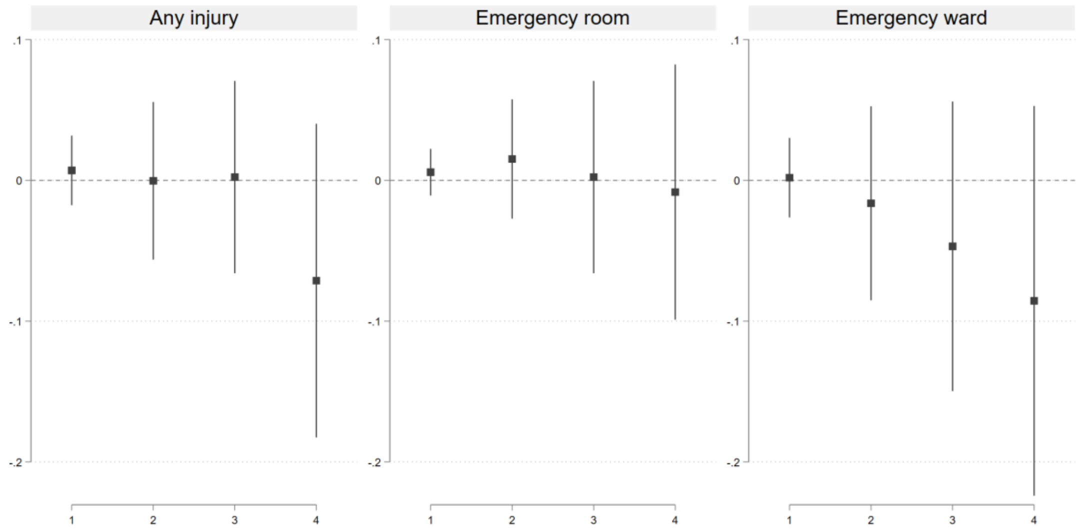

Figure S5. Reduced form. Associations between provider preference for ADHD medication and injuries in the general population sample. Coefficient plots with 95% confidence intervals based on linear probability models.

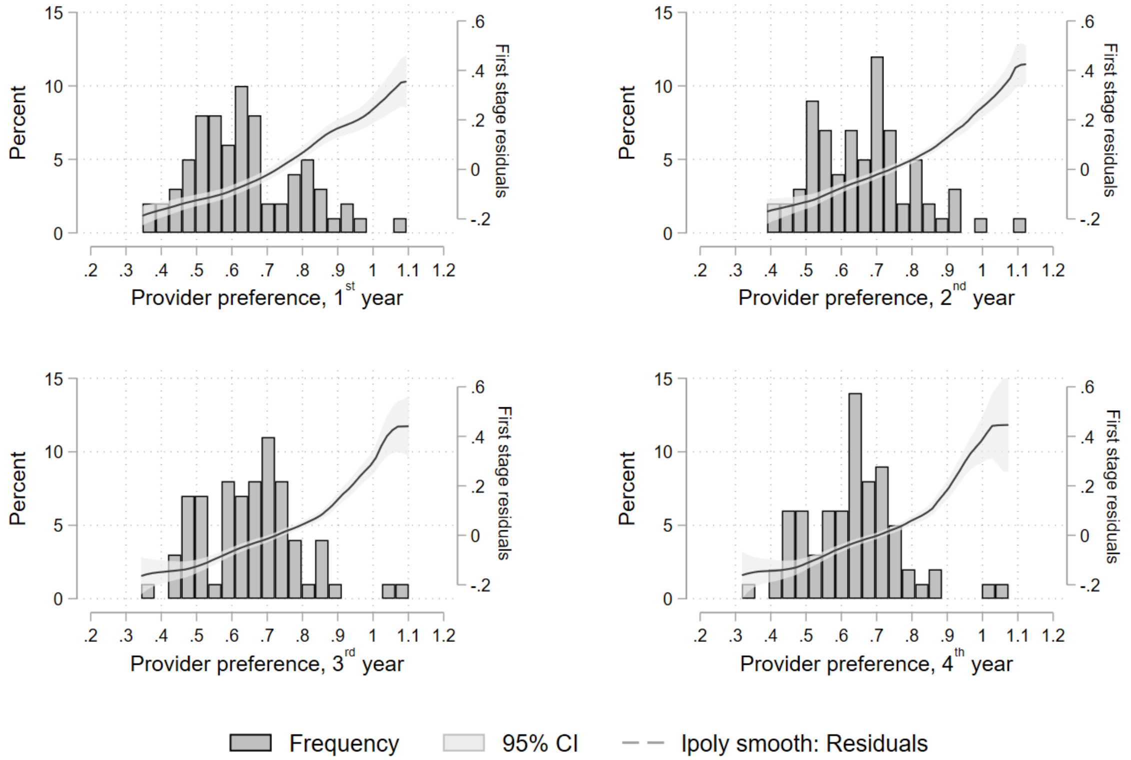

Figure S6. Variation between clinics in ADHD medication among patients diagnosed with ADHD for the first to fourth year following ADHD diagnosis. Provider preference for ADHD medication at clinic level as mean defined daily dosages for ADHD medication by years after ADHD diagnosis among patients on *x*-axis. Residuals from first stage regressions of treatment on IV plotted against values of IV with local polynomial regression line and residual values on right side *y*-axis.

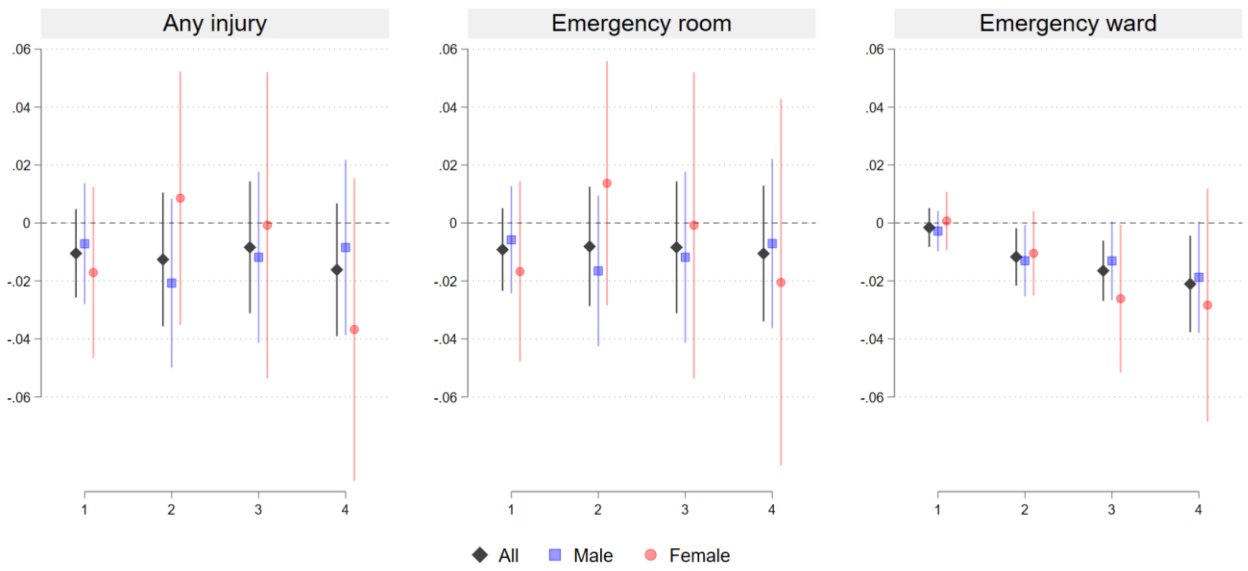

Figure S6. Probit results for association between ADHD medication and injuries.

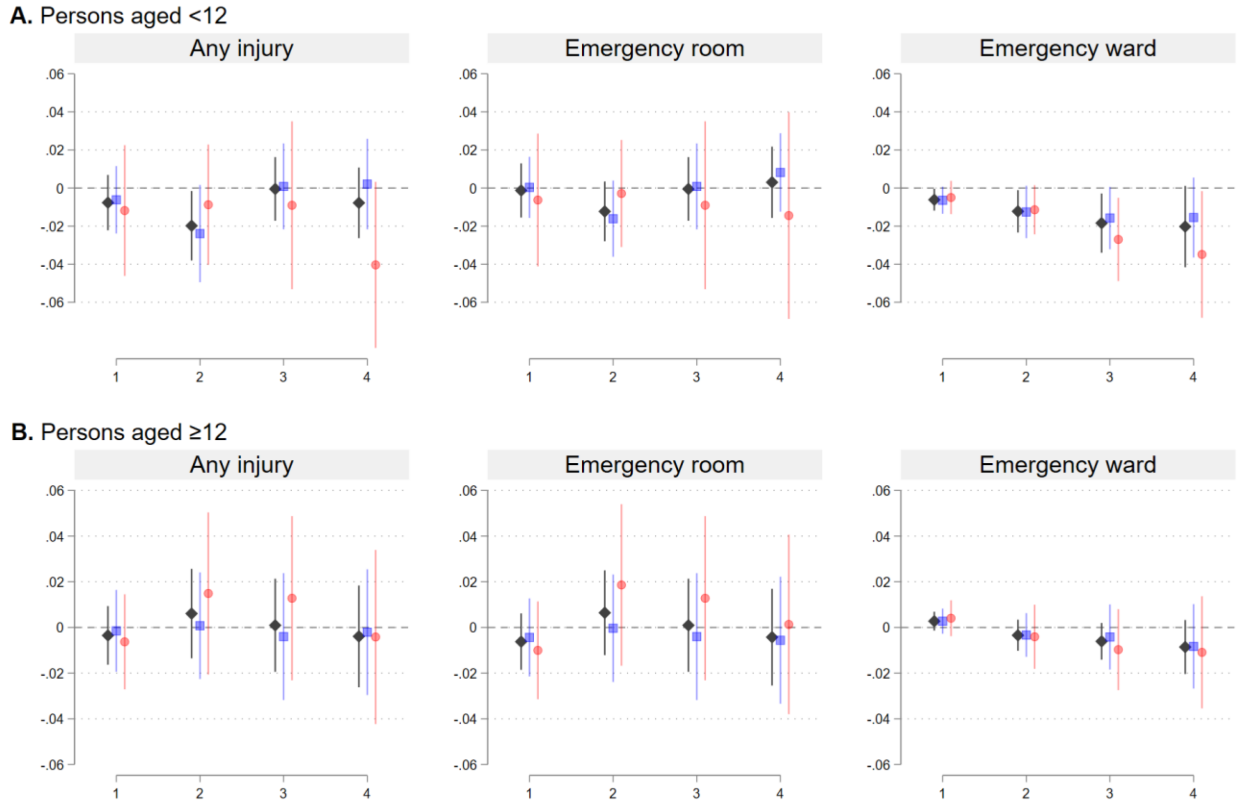

Figure S7. Linear probability model results for association between ADHD medication and injuries in patients aged below and above 12 years.

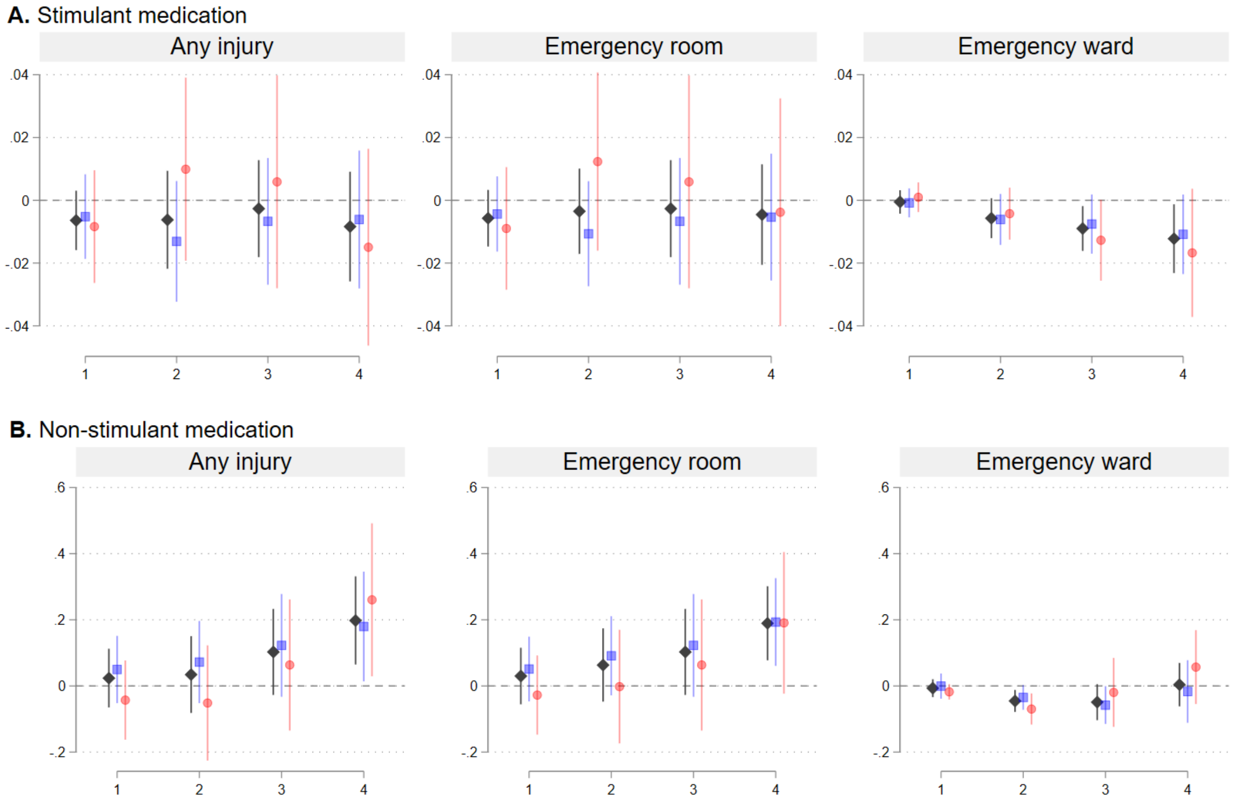

Figure S8. Linear probability model results for associations between ADHD medication and injury by type of medication.

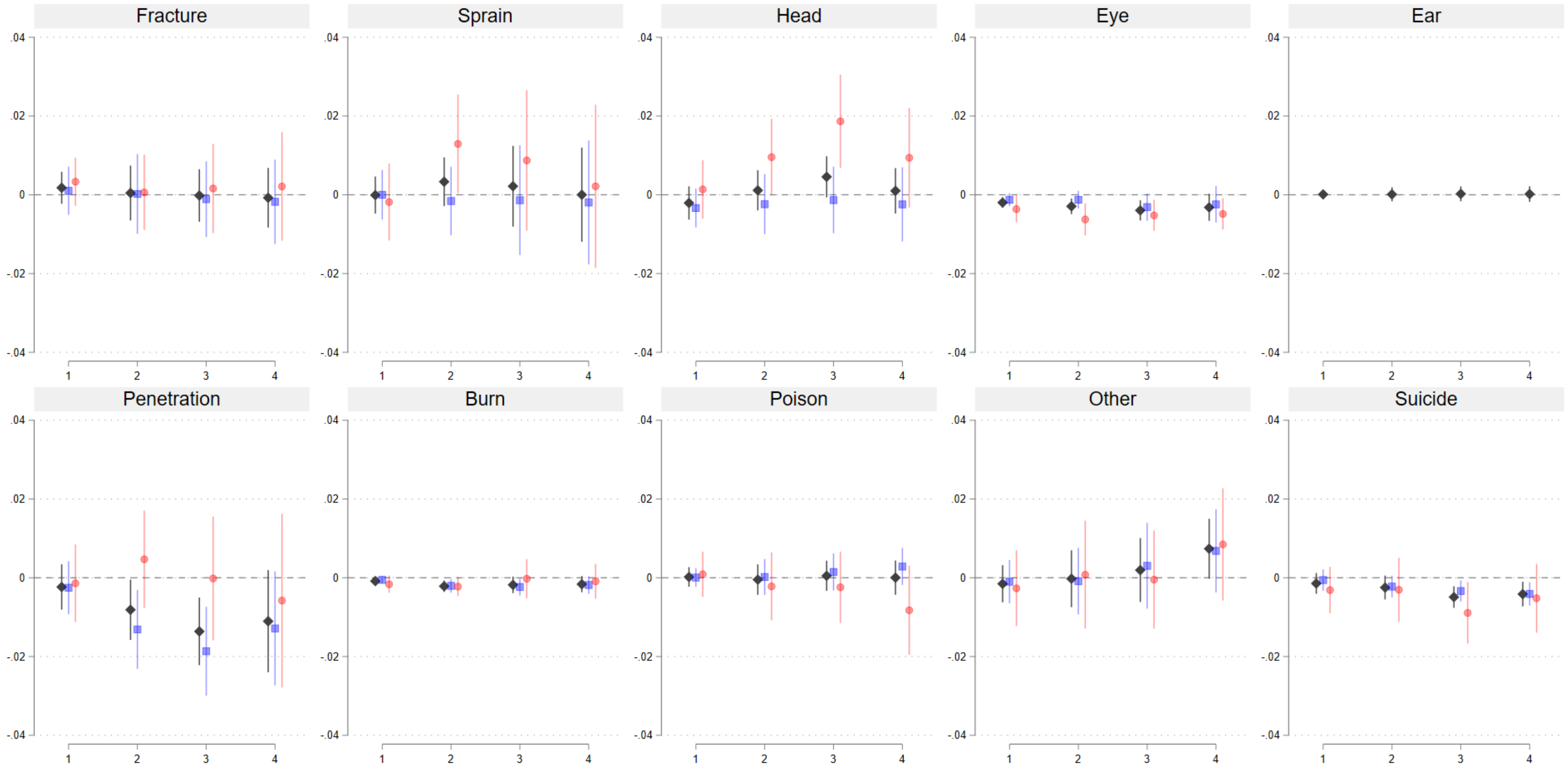

Figure S9. Linear probability model results for the association between ADHD medication and types of injuries.

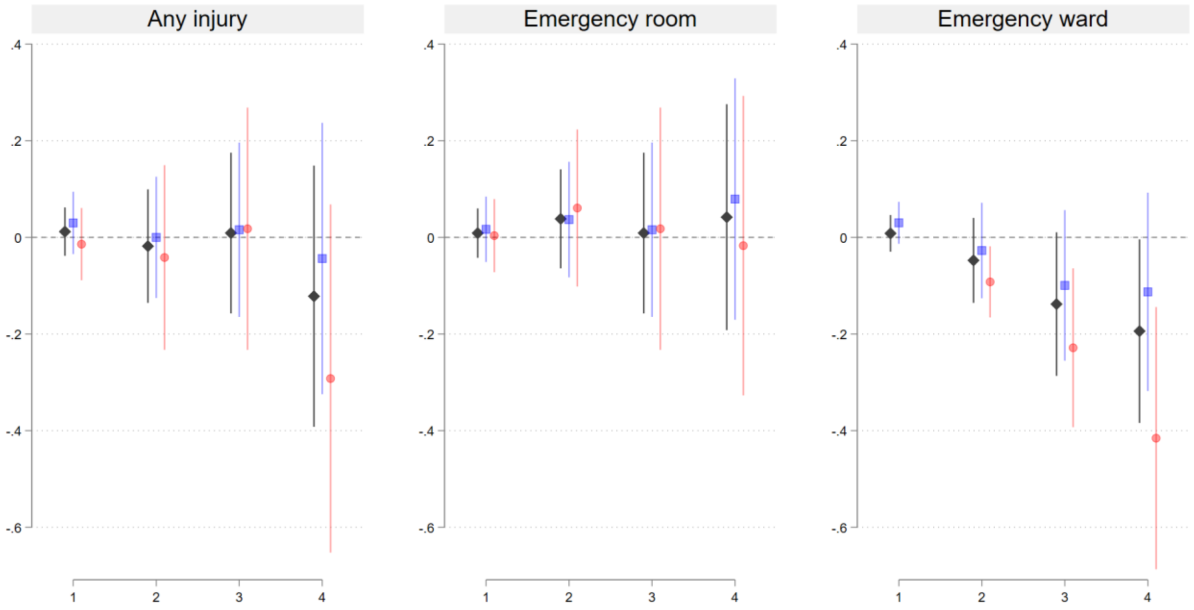

Figure S10. IV Probit results for the effect of ADHD medication on the probability of injury.

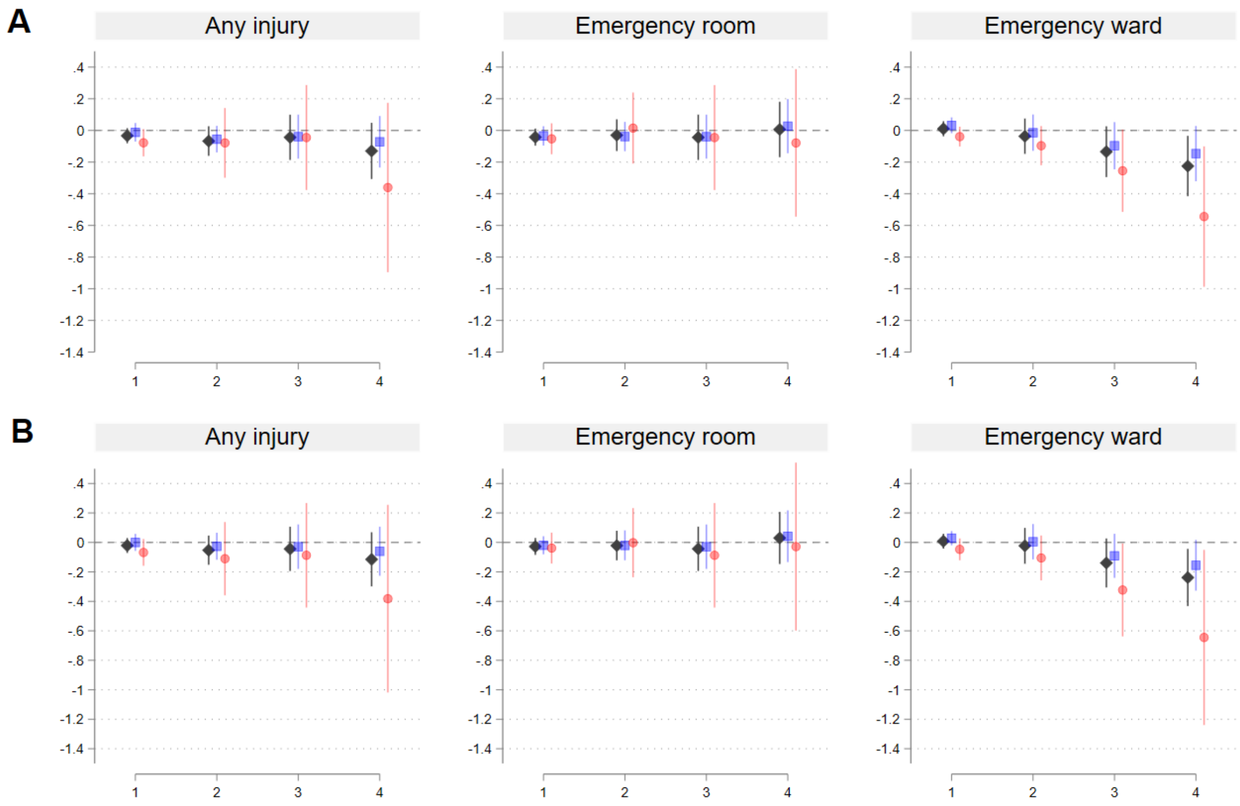

Figure S11. 2SLS results for the effect of ADHD medication on the probability of injury excluding patients who had one or more prescription prior to diagnosis. Panel A exclude patients with prescriptions prior to waitlist end date (sample n=6942). Panel B exclude patients with prescriptions prior to diagnosis (sample n=6,528).

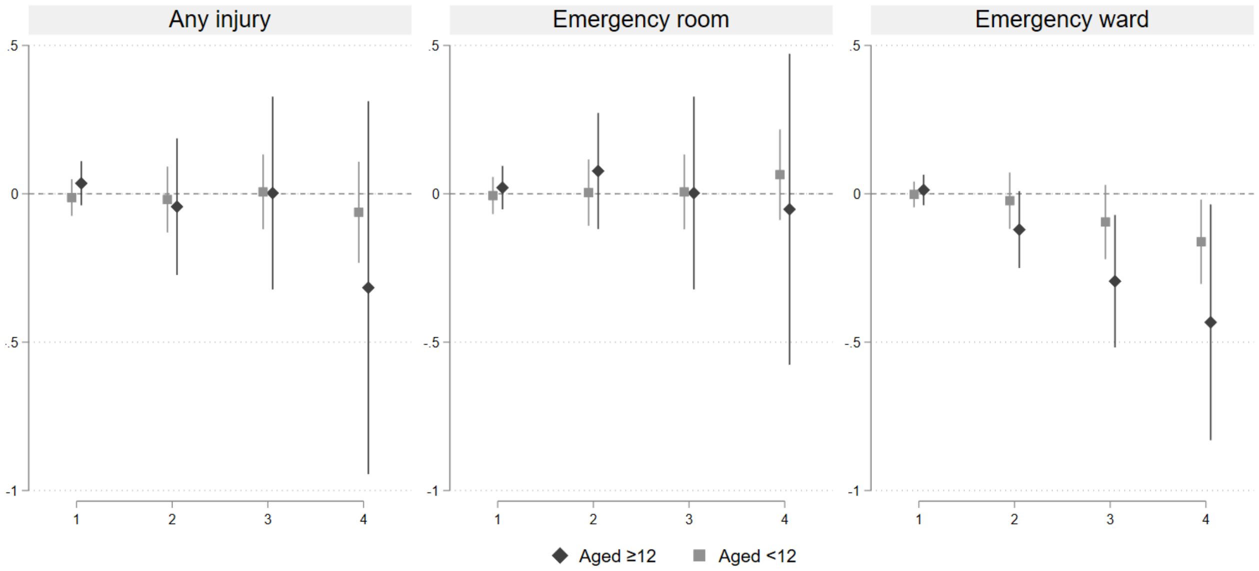

Figure S12. 2SLS results for the effect of ADHD medication on the probability of injury in patients aged below and above 12 years.

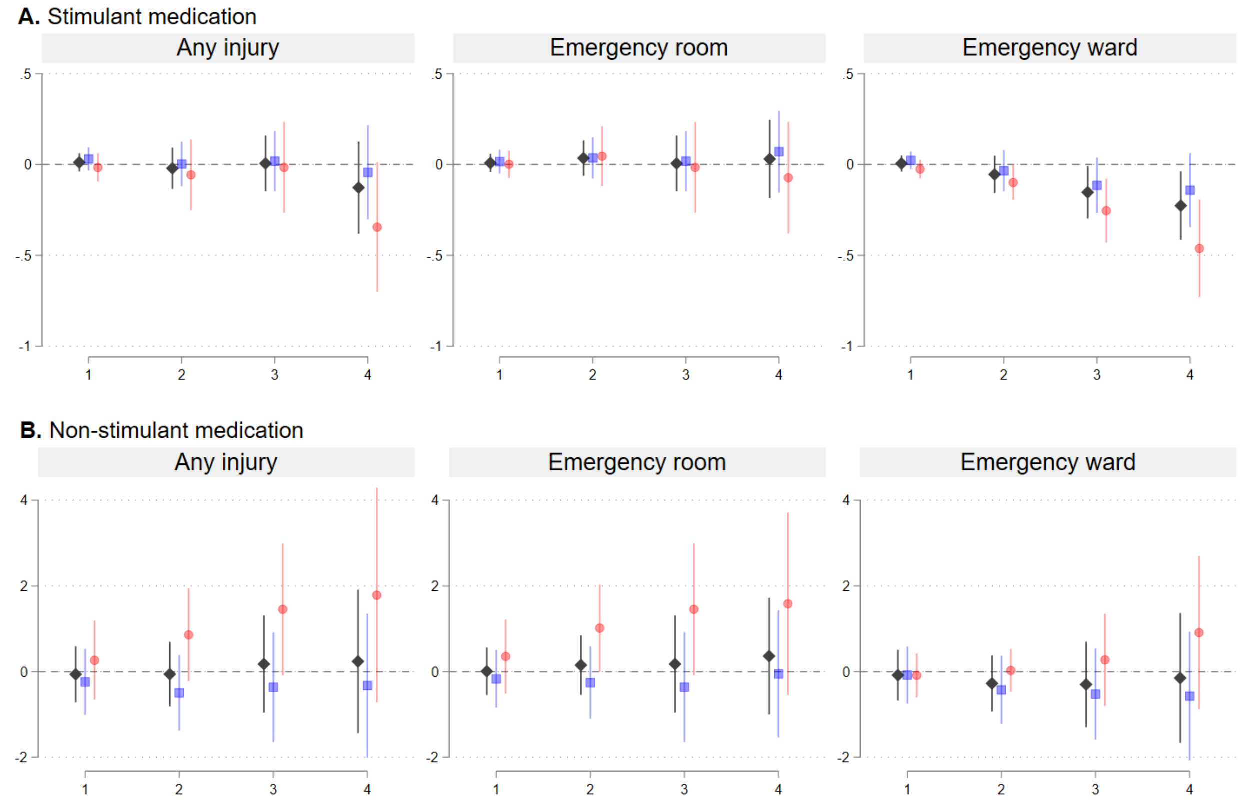

Figure S13. 2SLS results for the effect of ADHD medication on the probability of injury by type of medication.

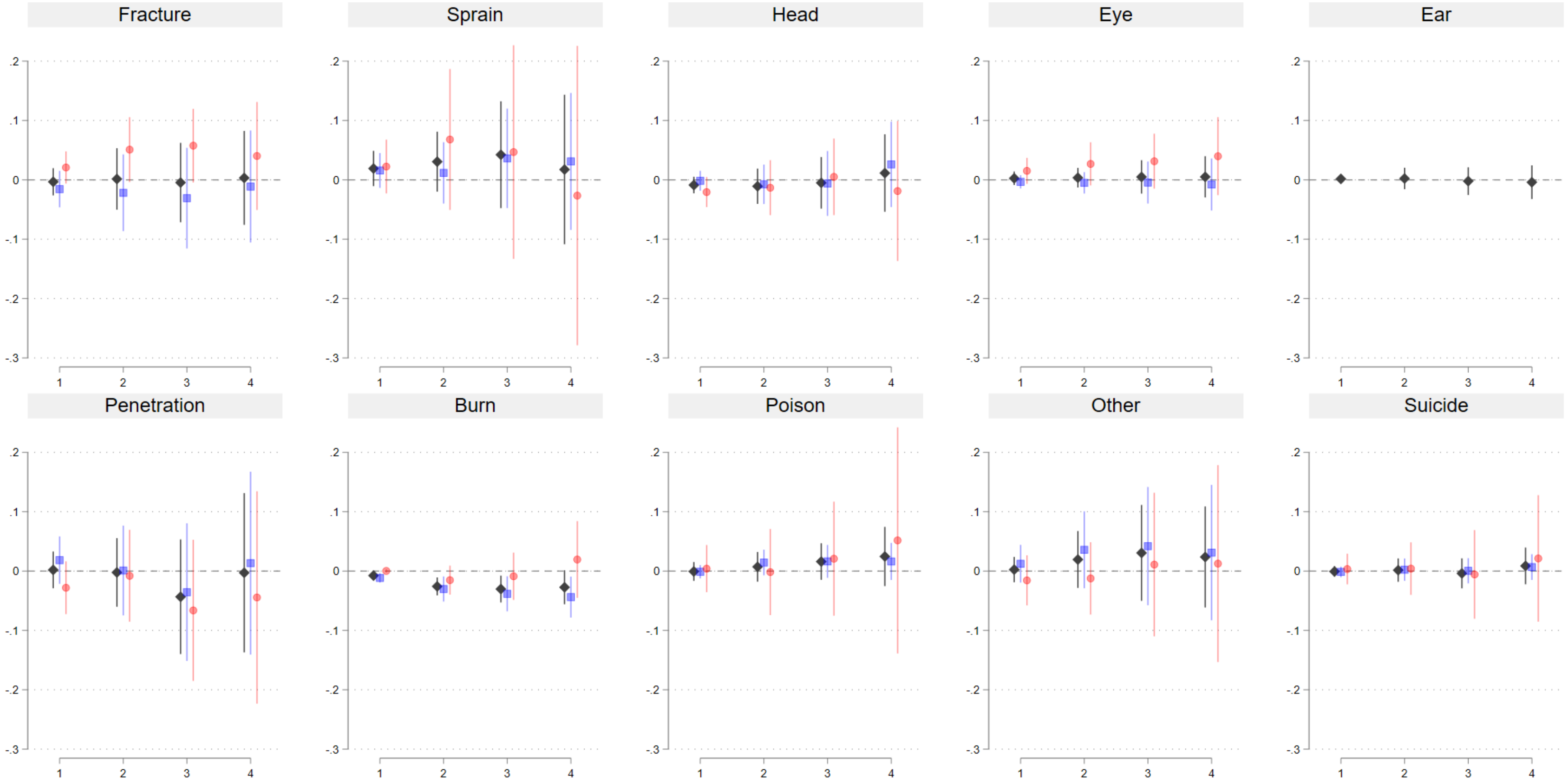

Figure S14. 2SLS results for the effect of ADHD medication on the probability of injury for types of injuries.

References

1. Widding-Havneraas T, Chaulagain A, Lyhmann I, et al. Preference-based instrumental variables in health research rely on important and underreported assumptions: a systematic review. *Journal of Clinical Epidemiology* 2021.

2. Glymour MM and Swanson AS. Instrumental Variables and Quasi-Experimental Approaches. In: Lash TL, VanderWeele TJ, Haneause S, et al. (eds) *Modern Epidemiology*. 4 ed. Wolters Kluwer, 2021, pp.677-709.

3. Hernán MA and Robins JM. *Causal Inference: What If*. Boca Raton: Chapman & Hall/CRC, 2020.
